# Implications of the unexpected persistence of human rhinovirus/enterovirus during the COVID‐19 pandemic in Canada

**DOI:** 10.1111/irv.12930

**Published:** 2021-11-07

**Authors:** David Champredon, Christina Bancej, Liza Lee, Steven Buckrell

**Affiliations:** ^1^ Public Health Risk Sciences Division, National Microbiology Laboratory Public Health Agency of Canada Guelph Ontario Canada; ^2^ Surveillance and Epidemiology Division, Centre for Immunization and Respiratory Infectious Disease Public Health Agency of Canada Ottawa Ontario Canada

**Keywords:** contact rate, respiratory viruses surveillance, rhinovirus, SARS‐CoV‐2/COVID‐19

## Abstract

Stringent public health measures imposed across Canada to control the COVID‐19 pandemic have nearly suppressed most seasonal respiratory viruses, with the notable exception of human rhinovirus/enterovirus (hRV/EV). Thanks to this unexpected persistence, we highlight that hRV/EV could serve as a sentinel for levels of contact rate in populations to inform on the efficiency, or the need of, public health measures to control the subsequent COVID‐19 epidemic, but also for future epidemics from other seasonal or emerging respiratory pathogens.

The 2019–2020 seasonal respiratory virus activity in Canada followed a typical trajectory until March 2020, when a series of stringent public health measures (PHM) aimed at controlling cases of COVID‐19 were imposed in many jurisdictions across Canada.^1^ The PHMs essentially reduced the effective transmission rate of SARS‐CoV‐2, but also of other pathogens transmitted by the respiratory route. Hence, in Canada and other countries,^2^, ^3^, ^4^, ^5^ the prevalence of most seasonal respiratory viruses (including influenza and respiratory syncytial virus [RSV]) ended abruptly in mid‐March and were virtually suppressed or well below historical levels throughout the 2020–2021 season in Canada (Figure S1). A notable exception were human rhinovirus/enterovirus (hRV/EV) infections which persisted through repeated stringent PHMs retaining, to some extent, their typical seasonal patterns in many Canadian jurisdictions.

The causes of hRV/EV persistence are not fully understood yet. The fact that hRV/EV are non‐enveloped may help sustain transmission thanks to their prolonged survival on surfaces.^6^ Masks may be less effective at stopping droplets and aerosols transporting hRV/EV.^7^ Another possibility may be that the population groups that drive hRV/EV transmission (typically young children) did not experience a contact rate reduction as effective as the groups driving SARS‐CoV‐2 transmission^8^ when social distancing and other PHMs were in place. Alternatively, hRV/EV transmissibility to and among children may be similar to or higher than that of SARS‐CoV‐2 in the pediatric population.^9^ Finally, the baseline prevalence of hRV/EV may be significantly higher than other respiratory infections (it also shows typical summertime persistence), allowing hRV/EV to rebound more easily when PHMs ease.

The persistence of hRV/EV highlights a new and unsuspected role for this family of viruses as a sentinel for the transmission rate for respiratory pathogens. Figure 1 suggests that, with the hindsight of 18 months of observations for both hRV/EV and SARS‐CoV‐2 infections in Canada, hRV/EV could have been used as a gauge for PHMs effectiveness as well as early warning for SARS‐CoV‐2 resurgences. Indeed, in four large provinces, where the signal is not too hampered by observation noise (hRV/EV is not reported by the province of Quebec, the second most populous Canadian province), the positivity rate of hRV/EV approximately mirrors, and often precedes by several weeks, the positivity rate for SARS‐CoV‐2 during the period when the vaccination coverage was null or low. Although the data to disentangle the intrinsic seasonality of hRV/EV infections and the effect of COVID‐19 PHMs are not available, it is conceivable that PHMs did impact hRV/EV detection rates, making them a potential early monitor of the efficacy of PHMs. When COVID‐19 vaccine coverage reached a substantial proportion of the population (starting in May 2021), a statistical analysis between SARS‐CoV‐2 and the lagged hRV/EV positivity rates suggests a decoupling of their incidence (Supporting Information); the latter are not vaccine‐preventable and thus followed their typical seasonality and intensity beyond May 2021.

**FIGURE 1:**
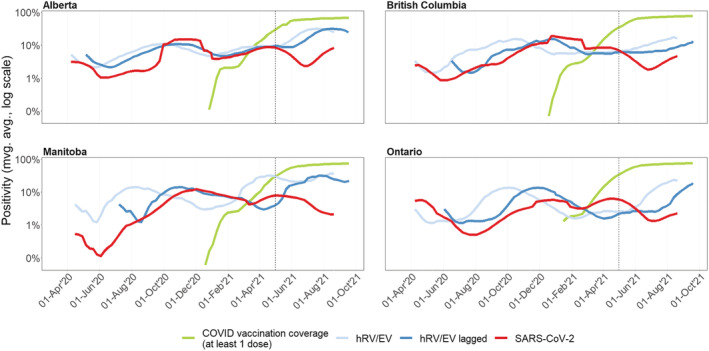
Test positivity for hRV/EV and SARS‐CoV‐2 in selected Canadian provinces. The positivity is defined as the number of tests with a positive result divided by the total number of tests performed to identify the corresponding viruses. The figure displays the 7‐day centered moving average of the positivity rate on a logarithmic scale (hRV/EV light blue, SARS‐CoV‐2 red). The dark blue line shows the lagged positivity rate for hRV/EV. The time lag is 4 weeks for Alberta, 10 weeks for British Columbia, 12 weeks for Manitoba, and 8 weeks for Ontario. The green curve represents the SARS‐CoV‐2 vaccine coverage for eligible individuals that have received at least one dose. The vertical dashed line indicates May 1, 2021, that separates the period when vaccine coverage is approximately above 30% in each province. Data sources: hRV/EV, Public Health Agency of Canada Respiratory Virus Detections Surveillance System (RVDSS, https://www.canada.ca/en/public‐health/services/surveillance/respiratory‐virus‐detections‐canada.html); SARS‐CoV‐2, PHAC COVID‐19/Canadian Network for Public Health Intelligence System for Analysing Laboratory Test counts (SALT); COVID‐19 vaccine coverage from https://health‐infobase.canada.ca/covid‐19/vaccination‐coverage/

The COVID‐19 pandemic has provided a real‐life experiment on how stringent PHMs on social distancing and enhanced personal protective measures (e.g., hand hygiene, respiratory etiquette, and face masks) impacted the circulation of seasonal respiratory viruses. Notably, it highlighted the unique role for hRV/EV incidence to gauge the contact rate of a population (most likely its younger age groups, where hRV/EV symptomatic infections may be more prevalent.^10^, ^11^ Direct measurements of contact rates through population surveys (for example, Drolet *et al* ^12^) are valuable but not sufficiently timely for surveillance, near‐term forecasting and thus for early warning of resurgence/re‐emergence potential for respiratory infectious diseases. Testing volumes for hRV/EV in Canada during the 2019–2020 and 2020–2021 seasons were higher than pre‐pandemic levels (Figure S4), suggesting potential different demographics tested during non‐pandemic seasons.

Our observations support the continuation for routine acute respiratory infections surveillance that includes hRV/EV and encourages enhancements (e.g., sampling and reporting across all ages and spectrum of illness). The current COVID‐19 pandemic has shown that monitoring the incidence of hRV/EV may have been undervalued so far: It could be useful for the subsequent SARS‐CoV‐2 epidemic activity and, for the longer term, potentially providing early warnings for other seasonal respiratory illnesses (notably influenza and RSV) as well as the transmission intensity at the time of introduction of another emerging respiratory pathogen in the monitored population.

## AUTHOR CONTRIBUTIONS

**David Champredon:** Conceptualization; writing‐original draft; formal analysis. **Christina Bancej:** Conceptualization; writing‐review and editing. **Liza Lee:** Data curation; writing‐review and editing. **Steven Buckrell:** Data curation.

### PEER REVIEW

The peer review history for this article is available at https://publons.com/publon/10.1111/irv.12930.

## Supporting information


**Figure S1.** Supporting Information

**Figure S2.** shows the estimates for the linear regression S~mR. The point and bar show the mean and 95%CI of the slope estimate m. The color indicates the time period where the regression was performed (orange for “pre”, green for “post”). The slopes for the “pre” period are all positive (p < 0.05 for all selected provinces) and changed to values closer to 0 for the “post” period, suggesting a decoupling of the lagged detection rates after May 2021 when vaccination increased to higher values (typically 70–80%, depending on provinces).
**Figure S3.** shows the scatter plot of the data the regression was performed on.

**Figure S4.** Supporting Information

## Data Availability

All data are publicly available.
