# Supplementary figures and images for: Implications of the unexpected persistence of human rhinovirus/enterovirus during the COVID‐19 pandemic in Canada
Source: Influenza Other Respir Viruses. 2021 Nov 7;16(2):190–2. doi: 10.1111/irv.12930 (PMC8652650; doi:10.1111/irv.12930)

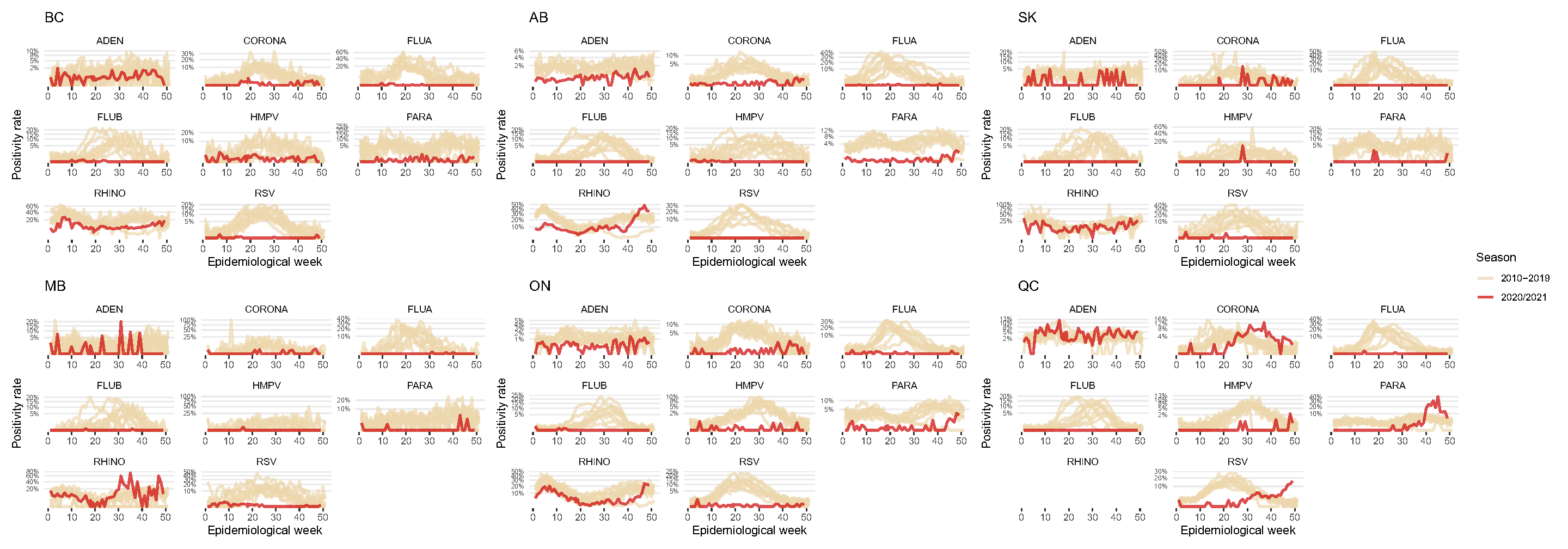

Supplement: Supplementary file 1 — Figure S1. Supporting Information [file IRV-16-190-s001.tiff]

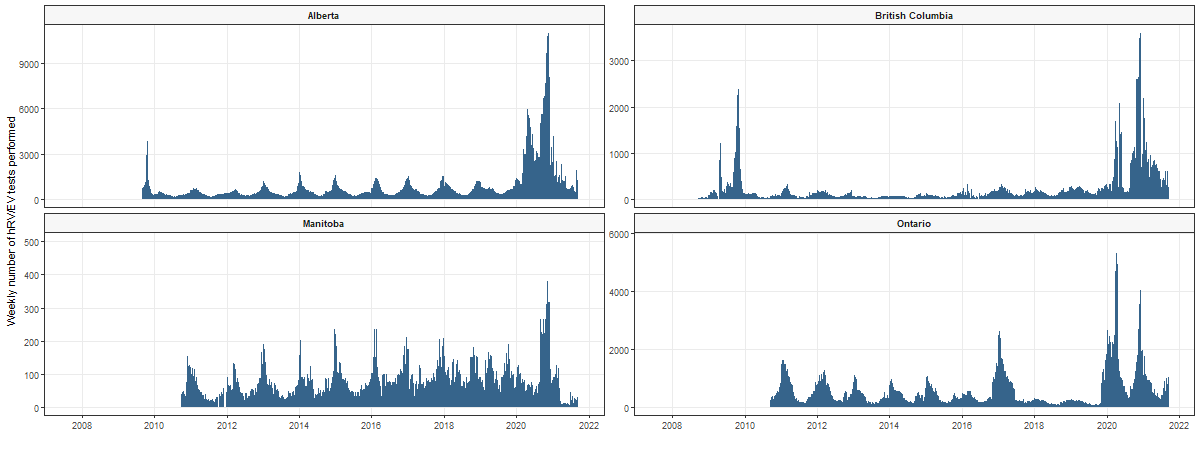

Supplement: Supplementary file 3 — Figure S4. Supporting Information [file IRV-16-190-s003.png]
