# Supplementary material for: Implications of the unexpected persistence of human rhinovirus/enterovirus during the COVID‐19 pandemic in Canada
Source: Influenza Other Respir Viruses. 2021 Nov 7;16(2):190–2. doi: 10.1111/irv.12930 (PMC8652650; doi:10.1111/irv.12930)

Supplemental Material – Statistical Analysis

We define $S$ as the weekly change of the 10-day moving average of SARS-CoV-2 positivity and $R$ the weekly change of the 10-day moving average of the *lagged* hRV/EV positivity. The value of the time lag depends on the province (see below for its determination).

To assess the association between the variations of detected COVID cases and hRV/EV cases, we perform a linear regression forcing the intercept at 0. In statistical term the regression formula is $S \sim mR$. The statistical analysis was conducted in R (version 4.0.2). A slope $m$ that is not statistically different from 0 would indicate a lack of support for an association between $S$ and$R$, whereas a value significantly different from 0 would support a relationship between both variables.

We divided the data set into two periods for this statistical analysis: the pre-vaccination period (“pre”) corresponding to data before May 1^st^, 2021 when the vaccine coverage (with at least on dose) was approximately below 30%, and the post-vaccination period (“post”) after this date.

For each province, the time lag for the variable $R$ was chosen to maximize the slope parameter $m$. We found the lag to be 4 weeks for Alberta (AB), 10 weeks for British Columbia (BC), 12 weeks for Manitoba (MB) and 8 weeks for Ontario (ON).

Figure S2 shows the estimates for the linear regression$S \sim mR$. The point and bar show the mean and 95%CI of the slope estimate $m$. The color indicates the time period where the regression was performed (orange for “pre”, green for “post”). The slopes for the “pre” period are all positive (p < 0.05 for all selected provinces) and changed to values closer to 0 for the “post” period, suggesting a decoupling of the lagged detection rates after May 2021 when vaccination increased to higher values (typically 70-80%, depending on provinces).

**Figure S2**


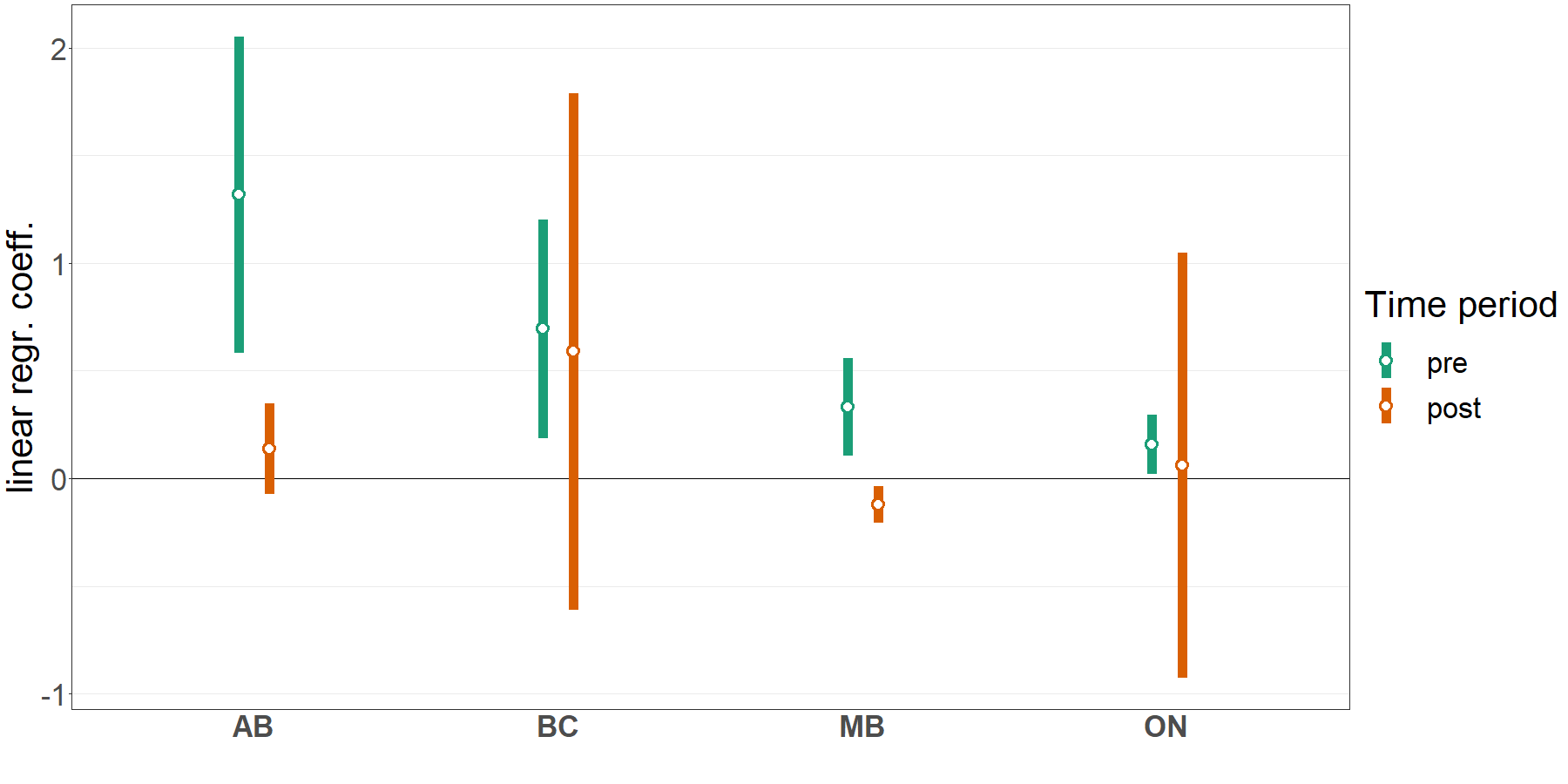


Figure S3 shows the scatter plot of the data the regression was performed on.

**Figure S3**


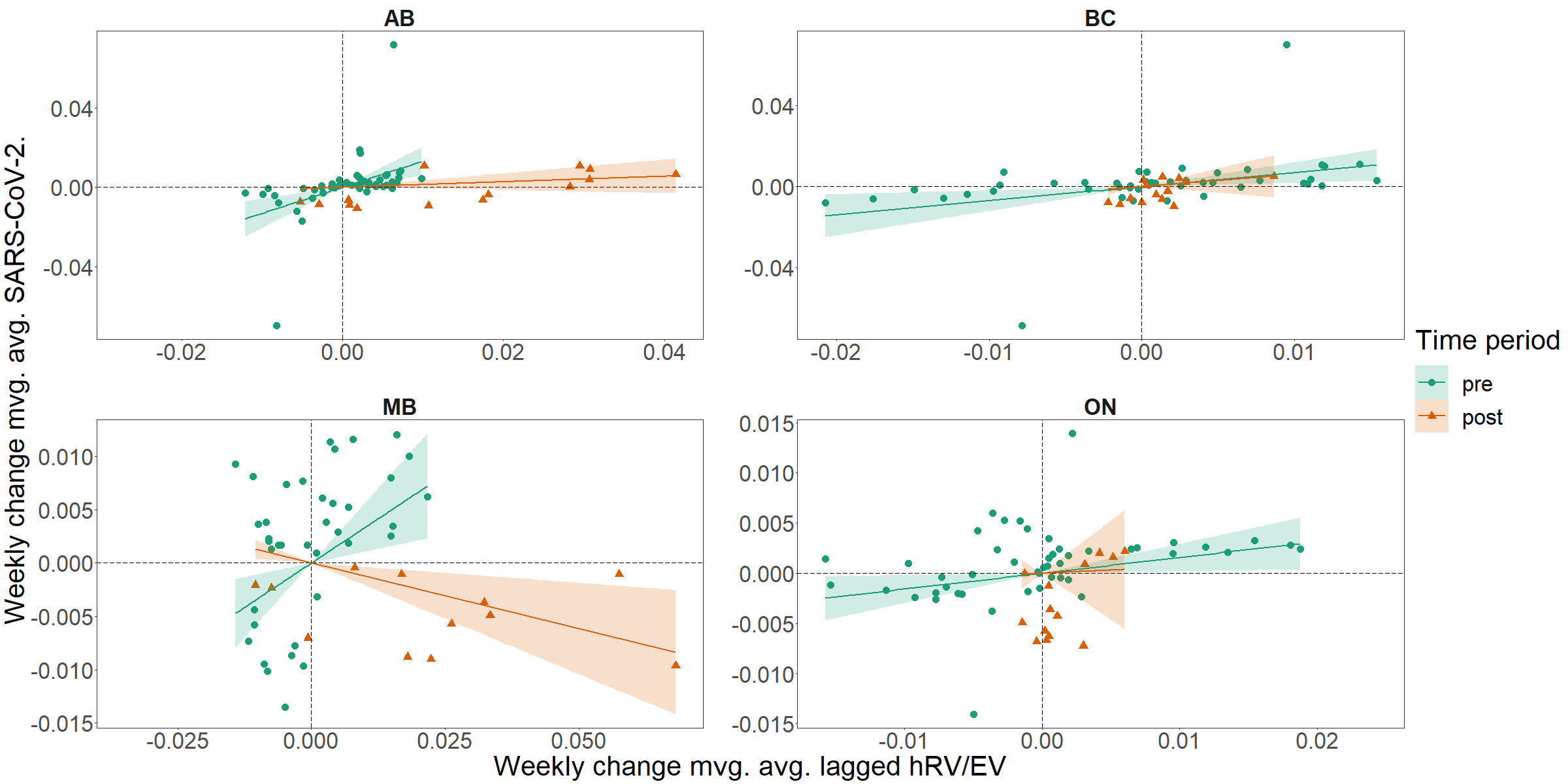

Supplement: Supplementary file 2 — Figure S2. shows the estimates for the linear regression S~mR. The point and bar show the mean and 95%CI of the slope estimate m. The color indicates the time period where the regression was performed (orange for “pre”, green for “post”). The slopes for the “pre” period are all positive (p < 0.05 for all selected provinces) and changed to values closer to 0 for the “post” period, suggesting a decoupling of the lagged detection rates after May 2021 when vaccination increased to higher values (typically 70–80%, depending on provinces). Figure S3. shows the scatter plot of the data the regression was performed on. [file IRV-16-190-s002.docx]
